# Supplementary material for: Tissue memory relies on stem cell priming in distal undamaged areas
Source: Nat Cell Biol. 2023 Apr 20;25(5):740–53. doi: 10.1038/s41556-023-01120-0 (PMC10185470; doi:10.1038/s41556-023-01120-0)
Supplement: Supplementary file 1 — Reporting Summary [file 41556_2023_1120_MOESM1_ESM.pdf]

nature portfolio

Corresponding author(s): Giacomo Donati

Last updated by author(s): Feb 1, 2023

Reporting Summary

Nature Portfolio wishes to improve the reproducibility of the work that we publish. This form provides structure for consistency and transparency in reporting. For further information on Nature Portfolio policies, see our Editorial Policies and the Editorial Policy Checklist.

Statistics

For all statistical analyses, confirm that the following items are present in the figure legend, table legend, main text, or Methods section.

n/a	Confirmed	
☐	☒	The exact sample size (*n*) for each experimental group/condition, given as a discrete number and unit of measurement
☐	☒	A statement on whether measurements were taken from distinct samples or whether the same sample was measured repeatedly
☐	☒	The statistical test(s) used AND whether they are one- or two-sided<br>*Only common tests should be described solely by name; describe more complex techniques in the Methods section.*
☐	☒	A description of all covariates tested
☐	☒	A description of any assumptions or corrections, such as tests of normality and adjustment for multiple comparisons
☐	☒	A full description of the statistical parameters including central tendency (e.g. means) or other basic estimates (e.g. regression coefficient) AND variation (e.g. standard deviation) or associated estimates of uncertainty (e.g. confidence intervals)
☐	☒	For null hypothesis testing, the test statistic (e.g. *F*, *t*, *r*) with confidence intervals, effect sizes, degrees of freedom and *P* value noted<br>*Give P values as exact values whenever suitable.*
☒	☐	For Bayesian analysis, information on the choice of priors and Markov chain Monte Carlo settings
☒	☐	For hierarchical and complex designs, identification of the appropriate level for tests and full reporting of outcomes
☐	☒	Estimates of effect sizes (e.g. Cohen's *d*, Pearson's *r*), indicating how they were calculated

Our web collection on statistics for biologists contains articles on many of the points above.

Software and code

Policy information about availability of computer code

Data collection	Leica Application Suite X (confocal microscopy)<br>BD FACS Verse, FACSAria II
Data analysis	Fiji (https://imagej.nih.gov/ij/)<br>Graphad prism software (GraphPad, version 7)<br>R studio<br>Seurat<br>Flow Jo

For manuscripts utilizing custom algorithms or software that are central to the research but not yet described in published literature, software must be made available to editors and reviewers. We strongly encourage code deposition in a community repository (e.g. GitHub). See the Nature Portfolio guidelines for submitting code & software for further information.

Data

Policy information about availability of data

All manuscripts must include a data availability statement. This statement should provide the following information, where applicable:

- Accession codes, unique identifiers, or web links for publicly available datasets
- A description of any restrictions on data availability
- For clinical datasets or third party data, please ensure that the statement adheres to our policy

Data reported in this paper will be shared by the lead contact upon request. This paper does not report original code. Any additional information required to reanalyse the data reported in this paper is available from the lead contact upon request. scRNA-seq, ATAC-seq and bulk RNA-seq data that support the findings of this study have been deposited in the Gene Expression Omnibus (GEO) under accession code GSE197590.

Human research participants

Policy information about studies involving human research participants and Sex and Gender in Research.

Reporting on sex and gender	Both sex were included in the analysis. No variance in different sexes would be assumed in this study.
Population characteristics	Cutaneous SCC samples with AK (UV-SCC) regions were obtained from 9 patients. In addition, cutaneous SCC samples were collected from 3 Recessive Dystrophic Epidermolysis Bullosa (RDEB) (EB-SCC) and 4 psoriasis patients (Pso-SCC), partecipant'ages ranged from 28 to 98.The RDEB patients harboured compound heterozygous mutations in COL7A1 (NM_000094.4) (patient 1: c.5443G>A (p.Gly1815Arg) and c.5819del (p.Pro1940Argfs*65), patient 2: c.5932C>T (p.Arg1978*) and c.8029G>A (p.Gly2677Ser), patient 3: c.7723G>A (p.Gly2575Arg) and c.8569G>T (p.Glu2857*))
Recruitment	Samples were prospectively collected from patients who agreed to participate in the study.
Ethics oversight	The institutional review board of the Hokkaido University Graduate School of Medicine approved the human study described (ID: 13-043, 14-063, and 15-029). The study was carried out according to the Declaration of Helsinki Principles. The participants provided written informed consent.

Note that full information on the approval of the study protocol must also be provided in the manuscript.

Field-specific reporting

Please select the one below that is the best fit for your research. If you are not sure, read the appropriate sections before making your selection.

☒ Life sciences ☐ Behavioural & social sciences ☐ Ecological, evolutionary & environmental sciences

For a reference copy of the document with all sections, see nature.com/documents/nr-reporting-summary-flat.pdf

Life sciences study design

All studies must disclose on these points even when the disclosure is negative.

Sample size	No statical method was used to determine the sample size prior to the study.
Data exclusions	No data were excluded.
Replication	Number of each replicates for each experiments is written in corresponding figures and figure legends.
Randomization	No randomization was done. Mice were categorised based on genotype.
Blinding	No blinding is done since the same researchers performed both data acquisition and analysis.

Reporting for specific materials, systems and methods

We require information from authors about some types of materials, experimental systems and methods used in many studies. Here, indicate whether each material, system or method listed is relevant to your study. If you are not sure if a list item applies to your research, read the appropriate section before selecting a response.

Materials & experimental systems

n/a	Involved in the study
☐	☒ Antibodies
☒	☐ Eukaryotic cell lines
☒	☐ Palaeontology and archaeology
☐	☒ Animals and other organisms
☒	☐ Clinical data
☒	☐ Dual use research of concern

Methods

n/a	Involved in the study
☒	☐ ChIP-seq
☐	☒ Flow cytometry
☒	☐ MRI-based neuroimaging

Antibodies

Antibodies used	Sca-1 (clone E13-161.7) (PE/Cyanine7 conjugated, Biolegend_122514), Glut1 (clone EPR3915) (Alexa Fluor 647 conjugated, Abcam_ab195020), CD45 (VioGreen, Miltenyi Biotec_130-110- 803), CD11b (clone M1/70) (FITC, Miltenyi Biotec_130-110-803), CD3 (clone 17.A2) (FITC, Miltenyi Biotec_130130-119-135), ydTCR (clone REA633) (PE-Vio770, Miltenyi Biotec_130-123-290), F4/80 (clone REA126) (PE-Vio770, Miltenyi Biotec_130-118-320), MHC-II (APC, Miltenyi Biotec_130-102-139), CD206 (clone C068C2) (PE, Biolegend_141706), IL-17A (PE, Biolegend_506903), cytokeratin 14 (clone LL002, Invitrogen), GM130 (clone 35, BD pharmingen), F4/80 (clone REA126, abcam_ab6640), anti-TNFa (clone MP6-XT22) (Brilliant violet 421 conjugated, biolegend_506327), Phospho-Histone H2A.X ((Ser139)CST_9718), SLC2A1 (GLUT1) (Sigma-Aldrich_ HPA031345), SLC2A1 (GLUT1) (clone EPR3915, abcam_ab115730), Ubiquityl-Histone H2A (Lys119) (clone D27C4, CST_8240), Keratin 6A (Biolegend_905701), anti-phospho-S6 Ribosomal Protein (Ser235/236) (clone D57.2.2E, CST_4858), anti-Ki67 (Abcam_16667), anti-E-cadherin (clone 24E10, CST_3195), anti-mFABP5 (RD System_AF1476).
Validation	All antibodies are well characterized and were applied according to data sheet instructions or previously published protocols.

Animals and other research organisms

Policy information about studies involving animals; ARRIVE guidelines recommended for reporting animal research, and Sex and Gender in Research

Laboratory animals	Rosa26-fl/STOP/fl-tdTomato (Madisen et al., 2010), Lgr5-EGFP-ires-CreERT2 (Jaks et al., 2008), Gata6-EGFP-ires-CreERT2 (Donati et al., 2017), Lrig1-EGFP-ires-CreERT2 (Page et al., 2013) and SKH-1 hairless mice were used.
Wild animals	Not applicable
Reporting on sex	Both sexes were used in this study, untless otherwise specified. For the scRNA-seq, female mice were selcted to avoid wounds from fighting in the cage.
Field-collected samples	Not applicable
Ethics oversight	Maintenance, care and experimental procedures have been approved by the Italian Ministry of Health, in accordance with Italian legislation (authorization no. 117/2018-PR), and the institutional review board of the Hokkaido University Graduate School of Medicine (authorization no. 22-0028).

Note that full information on the approval of the study protocol must also be provided in the manuscript.

Flow Cytometry

Plots

Confirm that:

☒ The axis labels state the marker and fluorochrome used (e.g. CD4-FITC).

☒ The axis scales are clearly visible. Include numbers along axes only for bottom left plot of group (a 'group' is an analysis of identical markers).

☒ All plots are contour plots with outliers or pseudocolor plots.

☒ A numerical value for number of cells or percentage (with statistics) is provided.

Methodology

Sample preparation	Whole skin: murine tail skin was collected, minced and incubated overnight in a collagenase D-pronase mixture to obtain single cell suspension. The suspension was then stained blocked with 3% FBS in PBS and then stained 30 min with the indicated dose of fluorophore-conjugated antibody.<br>Epidermal cells: murine tail skin was collected and epidermis isolated through overnight incubation with trypsin EDTA (0,25%). The epidermis was then minced and single cell suspension obtained for staining.
Instrument	FACS Verse and FACSAria II were used.

Software	FACS Diva and FlowJo (FlowJo™ v10.8) were used.
Cell population abundance	Final sorted populations made up of approximately 25% (Lrig1 GL), 4% (Gata6 GL) of 1% (Lgr5 GL) of tdTomato+, 5-10% of Lrig1 GL tdTomato+Sca1+, or 1-3% of Lrig1 GL tdTomato+Glut1+ of all cells. sc-RNAseq and bulk RNA-seq confirmed the identity of sorted cells.
Gating strategy	Gates were set to exclude debris and doublets: live cells (TOPRO-3/FSC-A), cell size (SSC-A/FSC-A), singlets (FSC-H/FSC-A). Gates on stained population were set on negative unstained controls and compensation was applied based on single-stained controls.

☒ Tick this box to confirm that a figure exemplifying the gating strategy is provided in the Supplementary Information.
